# ﻿*Torreyadapanshanica* (Taxaceae), a new species of gymnosperm from Zhejiang, East China

**DOI:** 10.3897/phytokeys.192.79506

**Published:** 2022-03-09

**Authors:** Yi-Fei Lu, Zi-Lin Chen, An-Guo He, Ju-Lian Liu, Pan Wang, Wei-Jie Chen, Xiao-Feng Jin

**Affiliations:** 1 State Key Laboratory of Subtropical Silviculture and School of Forestry and Bio-technology, Zhejiang A&F University, Hangzhou, Zhejiang 311300, China; 2 Zhejiang Provincial Key Laboratory of Forest Aromatic Plants-based Healthcare Functions, Zhejiang A&F University, Hangzhou, Zhejiang 311300, China; 3 College of Life Sciences, Zhejiang University, Hangzhou, Zhejiang, 310058, China; 4 Traditional Chinese Medicine Industry Development and Promotion Center of Pan’an County, Pan’an, Zhejiang 322300 China; 5 Administration of Zhejiang Dapanshan National Nature Reserve, Pan’an, Zhejiang, 322300, China; 6 Administration of Zhejiang Jiulongshan National Nature Reserve, Suichang, Zhejiang, 323300, China

**Keywords:** Gymnosperm, new species, *Torreyadapanshanica*, Zhejiang

## Abstract

*Torreyadapanshanica* X.F.Jin, Y.F.Lu & Zi L.Chen, a new species endemic to central Zhejiang, East China, is described and illustrated. This new species is most similar to *T.jiulongshanensis* (Z.Y.Li, Z.C.Tang & N.Kang) C.C.Pan, J.L.Liu & X.F.Jin, but differs in having leaves with an acuminate apex (vs. leaves with an acute apex), broadly ovoid-globose or globose seeds (vs. obovoid to narrowly obovoid seeds), slightly emarginate at the apex and obtuse-rounded at the base (vs. both acute at the apex and base), testa with irregular shallow grooves (vs. testa smooth or sometimes slightly concave). The diagnostic characters are critically compared and an IUCN assessment for the risk to the new species is estimated.

## ﻿Introduction

*Torreya* Arn. (Taxaceae), containing eight species, is distributed in E Asia and SE to W America (Yang et al. 2017). The genus is represented by five species native to China together with *Torreyanucifera*, a cultivated species. *Torreyagrandis* is both widely distributed and cultivated in East China (Cheng et al. 1978; Fu et al. 1999; Teng et al. 2017).

Female gametophyte tissue of seeds in cultivated *Torreyagrandis* can be produced as edible ‘nuts’ and are called ‘Xiāngfěi’ in Chinese, which are available for sale in the markets in Zhejiang Province. In Zhejiang, as Ching reported in Hu (1927), people in Shengxian [Shen Hsien in the Wade-Giles Romanization system of writing Chinese], Kuaiji [Kwei-che], and Zhuji [Chu-che Hsien] have long since been meeting to sell nuts, although the region is very rugged, making traveling and transportation very difficult at that time. The ‘Xiāngfěi,’ fried seeds of the cultivated varieties viz. *T.grandis* ‘Merrillii’, produced from Fengqiao in Zhuji County are the most famous now for their large seeds, high yield, and good quality (Teng et al. 2017).

The species *Torreyagrandis* is widely cultivated in the central to southwestern regions of Zhejiang, and some cultivars had long been recognized (Hu 1927). Chun (1925) described a long-leaved species, *T.jackii*, which was collected by Ching from Xianju, Zhejiang. Hu (1927) conducted a taxonomic study on Chinese *Torreyas*, and recognized three native species, placing *Torreyafargesii* and *T.jackii*, which have deeply ruminate megagametophyte (female gametophyte tissue) in the sect. Ruminatae, while *T.grandis* with slightly ruminate megagametophyte was placed in sect. Nuciferae. Based on the variable size and shape of seeds of *T.grandis*, Hu recognized and described four varieties and one form from Fengqiao, Zhuji County; these infraspecific taxa were later recognized as cultivated varieties of *T.grandis* (Zhang 1993). Cheng et al. (1975) described *Torreyayunnanensis* as a new species from NW Yunnan, which was later classified as a variety, T.fargesiivar.yunnanensis, by Kang (Kang and Tang 1995). Another new species, *Torreyaparvifolia* described from S Sichuan, is similar to *T.yunnanensis* but differs in having shorter leaves and smaller seeds (Yi et al. 2006). Kang and Tang (1995) reviewed the taxonomy of the genus *Torreya*, and described a new variety, T.grandisvar.jiulongshanensis, from Suichang County of SW Zhejiang. This variety has longer leaves and deeply ruminate megagametophyte, and was recently treated as an independent species (Teng et al. 2017). *Torreyajiulongshanensis* is at variance with the broader species concepts of *Torreyagrandis* by Farjon (2010) and Eckenwalder (2009). These authors consider it as a variety or synonym of *Torreyagrandis*.

During preparation of the new version of the “Flora of Zhejiang”, while developing a plan to protect the extremely small population of *Torreyajiulongshanensis*, an unusual *Torreya* was collected from Pan’an in central Zhejiang, which is similar to *T.jiulongshanensis*, but differs in having larger seeds, relatively longer leaves and acuminate apex (Teng et al. 2017). A precise morphological comparison of leaves and seeds revealed this taxon as a distinct new species, which we name and describe below.

## ﻿Results

### ﻿ Torreya dapanshanica

Taxon classificationPlantaePinalesTaxaceae

X.F.Jin, Y.F.Lu & Zi L.Chen sp. nov.

70547570-5169-5861-BE75-8BA8E1AAC094

urn:lsid:ipni.org:names:77295798-1

Figs 1 , 2

#### Latin diagnosis.

*Species nova haec* T. jiulongshanensi *(Z.Y.Li et al.) C.C.Pan et al. affinis est, sed a qua foliis apice acuminatis, basi cuneatis, seminibus late ovoideo-globosis vel globosis, basi obtusis, testis irregulariter et vadose canaliculatis differt*.

#### Type.

**China.** Zhejiang: Pan’an, Mount Dapanshan, Huaxi, on slope under forest, 28°58'37.41"N, 120°30'00.01"E, alt. 420 m, 22 August 2017, *Xiao-Feng Jin* 4036B (holotype: ZM; isotypes: HTC, PE, ZJFC, ZM).

***Trees*** evergreen, 5‒8 m tall, with trunk to 25 cm d.b.h., dioecious; bark gray-brown, irregularly vertically fissured; branches glabrous, slightly shiny, with young branches green and biennial ones yellow-green or green. ***Leaves*** decussate, base coiled and 2-ranked; blade linear, (1.9‒)3‒5(‒6.9) cm long, 2.7‒3.2 mm wide, upper part slightly falcate, apex acuminate and spiculate, base cuneate, adaxially dark green, shinning, with mid-rib slightly concave, 2 grooves from base to near apex, abaxially green, with mid-rib slightly raised, 2 stomatal bands brown, each nearly equal to mid-rib in width, marginal band ca. 2× as wide as stomatal band; petioles short, ca. 1 mm long, yellow-brown. ***Pollen cones*** solitary, axillary, ovoid-globose, 9‒12 mm long, 7‒9 mm wide, base shortly pedunculate; peduncles 3‒3.5 mm long; bracts 5 or 6-pairs, decussate, abaxially ridged, lowermost 2 pairs smaller, green, papery, others yellow-green, thinly papery; microsporophylls 42‒56, spiral in 6‒8 whorls, triangular-ovate, membranous, 2‒2.5 mm long, apex praemorse with 9 minute teeth, each with 4 pollen sacs abaxially; pollen sac yellow, ellipsoid, 1.2‒1.5 mm long, ca. 0.8 mm wide, longitudinally divided. ***Seed-bearing structures*** borne in pairs in leaf axils, sessile, 6‒7 mm long, each with 2 pairs of decussate bracts and 1 lateral bract; bracts sub-leathery, abaxially ridged. ***Aril*** succulent, base with persistent bracts; seed (including aril) obovoid, 3.5‒4 cm long, 2‒2.5 cm in diam., apex slightly convex with a mucro, seed (excluding aril) broadly ovoid-globose or globose, apex slightly emarginate, base obtuse-rounded; testa ligneous, stiff, with irregular shallow grooves; female gametophyte tissue deeply ruminate.

**Figure 1.**
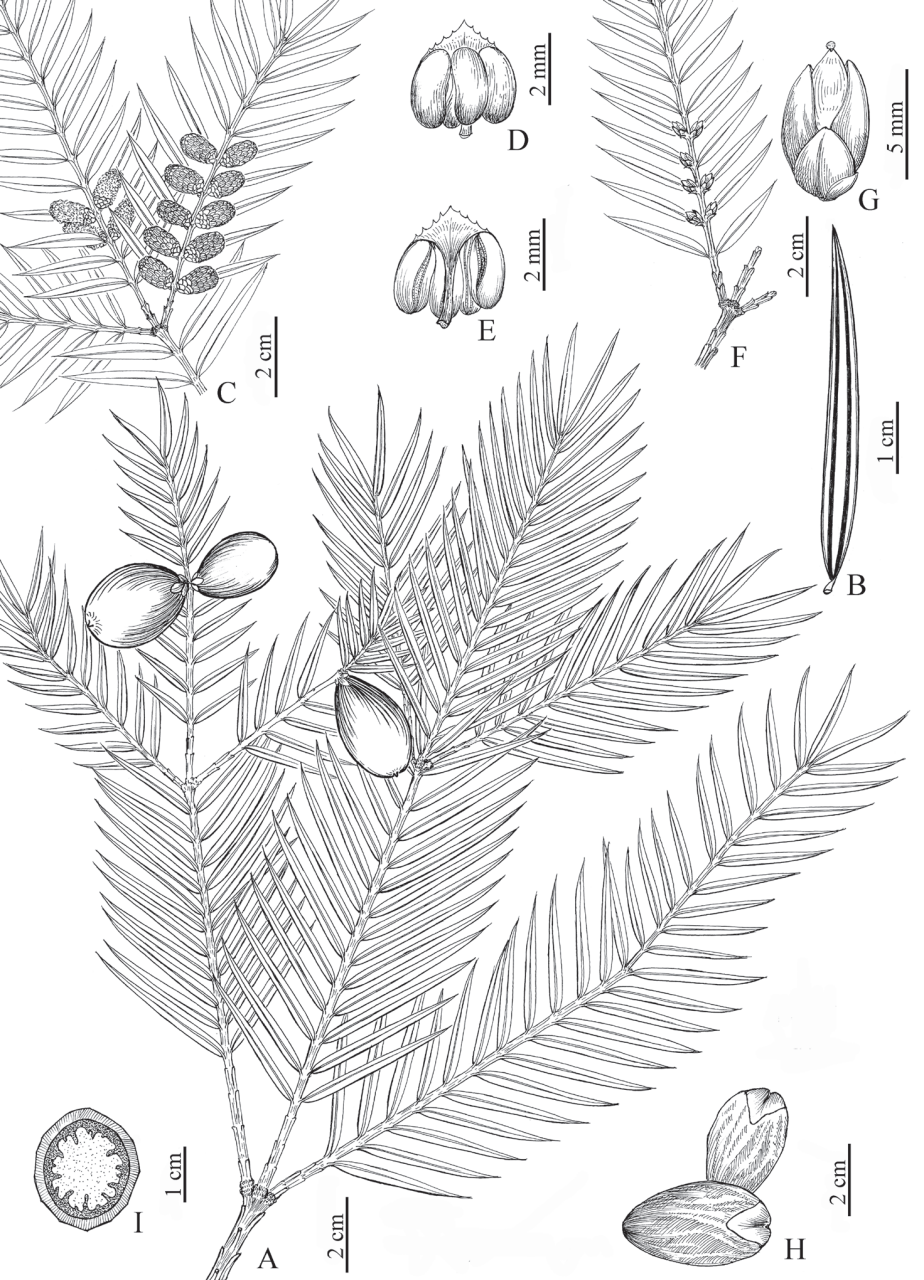
*Torreyadapanshanica* sp. nov. **A** branch with seeds **B** leaf (abaxial surface) **C** branch with pollen cones **D** microsporophyll/stamen (abaxial surface with four pollen sacs) **E** microsporophyll/stamen (adaxial surface) **F** branch with ovules **G** ovule (showing macrosporophyll and bracts) **H** seeds without aril **I** cross section of seed (showing deeply ruminate megagametophyte) (drawn by Xiao-Feng Jin; based on *Xiao-Feng Jin* 4036B, ZM).

#### Distribution and habitat.

This new species is known only from Mount Dapanshan of Pan’an County, central Zhejiang. It grows at a single location on a forested slope by a stream margin at an elevation of 420‒485 m.

**Figure 2.**
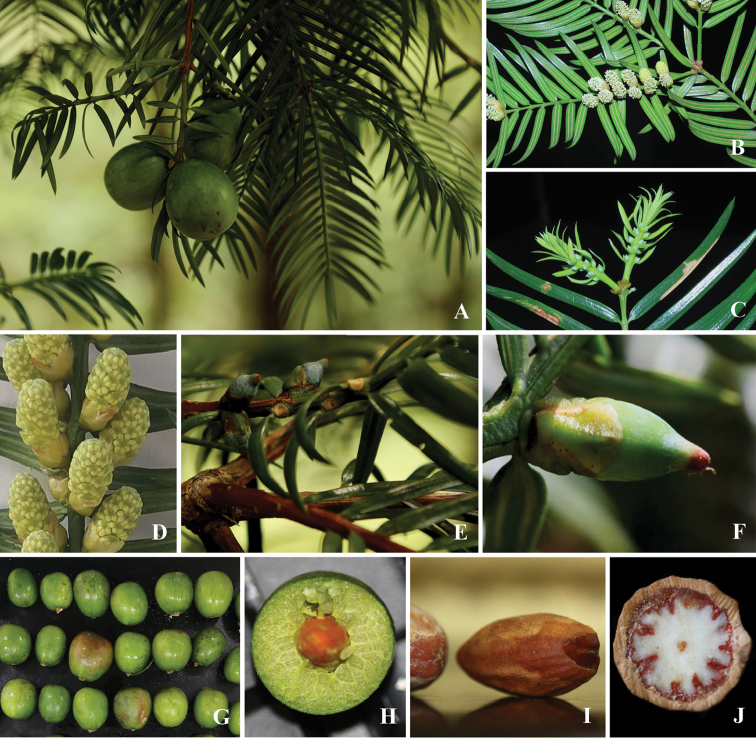
Photographs of *Torreyadapanshanica* sp. nov. **A** branch with seeds **B** branch with pollen cones **C** branch with pollen cones **D** pollen cone showing pollen sacs **E** ovule (fertilized) **F** fertilized ovule with bracts **G** seeds with arils **H** cross section of seed (showing aril) **I** seeds without aril **J** cross section of seed (showing deeply ruminate megagametophyte).

#### Phenology.

Pollen cones observed from late early July to early the following April; ovules from mid-November to late the following April. Seeds mature from September to October.

#### Etymology.

The specific epithet ‘*dapanshanica*’ refers to the type locality of the new species.

#### Conservation status.

Critically Endangered (CR) [B2ab(ii)D] (International Union for Conservation of Nature, IUCN 2019). The new species is only known from the type locality, Mount Dapanshan in Pan’an County, and occupies less than 1 km^2^ with about six mature individuals and two seedlings. Moreover, the habitat destruction and seed harvesting by local people still exist. This species is considered as Critically Endangered (CR) according to classification methods used by the IUCN Red List Categories and Criteria (IUCN 2019) based on the current survey.

#### Specimen examined.

Zhejiang: Pan’an, Mount Dapanshan, Huaxi, in forest by stream, 28°58'37.41"N, 120°30'00.01"E, alt. 470 m, 24 November 2012, *Xiao-Feng Jin & Ying-Ying Zhou* 2938 (ZJFC, ZM), alt. 479 m, 24 November 2012, *Xiao-Feng Jin & Ying-Ying Zhou* 2940 (ZM); the same locality, on slope by stream, 28°58'33.39"N, 120°30'02.06"E, alt. 485 m, 7 April 2013, *Xiao-Feng Jin* 2953 (ZM), 25 July 2016, *Xiao-Feng Jin* 3831 (ZM), 3832 (ZM), 22 August 2017, *Xiao-Feng Jin* 4038B (ZM), 4039B (ZM); the same locality, in forest, alt. 450 m, 29 March 2020, *Xiao-Feng Jin & Yi-Fei Lu* 4547 (ZM), alt. 480 m, 29 March 2020, *Xiao-Feng Jin & Yi-Fei Lu* 4548 (ZM).

#### Comparison.

**Figure 3.**
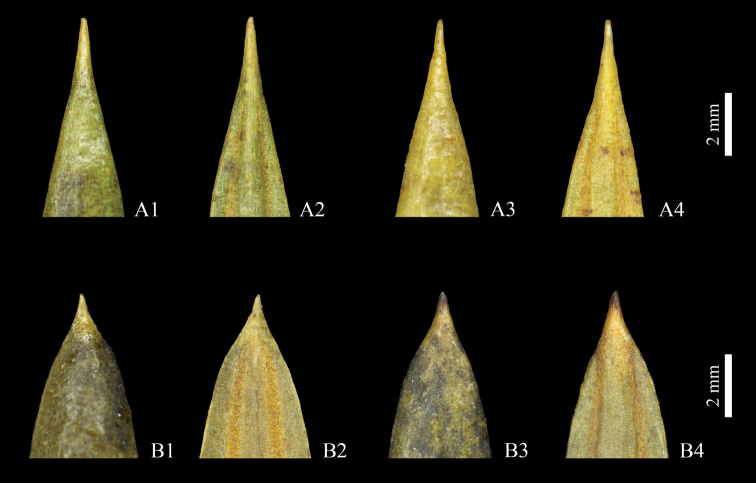
Leaf apex of *Torreyadapanshanica* (**A1‒A4**) and *T.jiulongshanensis* (**B1‒B4**) **A1** and **B1** seed bearing individuals (adaxially) **A2** and **B2** seed bearing individuals (abaxially) **A3** and **B3** pollen cone bearing individuals (adaxially) **A4** and **B4** pollen cone bearing individuals (adaxially).

Teng et al. (2017) analyzed the leaf variation of all wild species of *Torreya* from Zhejiang, and found the leaves of T.grandisvar.jiulongshanensis were different from those of *T.grandis* and *T.jackii*. Consequently, var. jiulongshanensis was treated as an independent species and combined as *T.jiulongshanensis*. Within some populations identified as *T.jiulongshanensis*, Teng et al. (2017) mentioned that the leaves from the population in Pan’an were different from the others. Herein, we compared the leaf apex, and found that those from Pan’an (*T.dapanshanica*) are acuminate, whereas those from Suichang (*T.jiulongshanensis*) are acute (Fig. 3).

**Figure 4.**
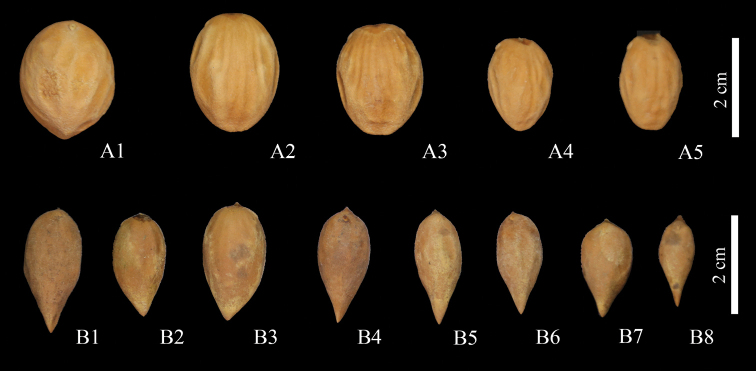
Variation of seed shape and size of *Torreyadapanshanica* (**A1‒A5**) and *T.jiulongshanensis* (**B1‒B8**).

Seed morphology of *Torreyadapanshanica* is also different from *T.jiulongshanensis* (Fig. 4). The seeds of *Torreyadapanshanica* are broadly ovoid-globose or globose, 25.52±3.52 mm × 15.62±3.67 mm, apex slightly emarginate, base obtuse-rounded, testa with irregular shallow grooves. *Torreyajiulongshanensis* has seeds that are obovoid to narrowly obovoid, 22.56±3.28 mm × 10.29±2.23 mm, both apex and base acute, testa smooth or sometimes slightly concave.

A key to all known species of *Torreya* from China is shown below.

### ﻿Key to the species of *Torreya* from China

**Table:** 

1	Female gametophyte tissue slightly ruminate	***T.grandis***
–	Female gametophyte tissue deeply ruminate	**2**
2	Inner wall of seed coat smooth; female gametophyte tissue without grooves	**3**
–	Inner wall of seed coat with 2 opposite longitudinal ridges; female gametophyte tissue with 2 conspicuous longitudinal grooves	**6**
3	Leaves 1.2‒3 cm long, usually straight	***T.fargesii***
–	Leaves 2.5‒7 cm long, slightly to strongly falcate	**4**
4	Leaves 2.5‒5 cm long, stomatal bands brown; arils green-brown or brown, not powdery	**5**
–	Leaves 3.5‒7 cm long, stomatal bands silvery gray; arils white powdery	***T.jackii***
5	Leaf apex acute; seeds obovoid or narrowly obovoid, base acuminate; testa usually smooth	***T.jiulongshanensis***
–	Leaf apex acuminate; seeds broadly ovoid-globose or globose, base obtuse; testa with irregular and shallow grooves	***T.dapanshanica***
6	Leaves 2‒3.6 cm long; testa smooth	***T.yunnanensis***
–	Leaves 1.2‒2 cm long; testa with irregular and shallow grooves	***T.parvifolia***

## Supplementary Material

XML Treatment for Torreya dapanshanica
